# Flexural Properties and Failure Mechanisms of Short-Carbon-Fiber-Reinforced Polylactic Acid Composite Modified with MXene and GO

**DOI:** 10.3390/ma17061389

**Published:** 2024-03-18

**Authors:** Xu Wang, Shao-Cong Li, Duo-Wen Xiang, Min Gao, Hong-Mei Zuo, Dian-Sen Li

**Affiliations:** 1 School of Textile and Garment, Anhui Polytechnic University, Wuhu 241000, China; lsc1419463244@163.com (S.-C.L.); xdw040103@163.com (D.-W.X.); wy_zsh2022@163.com (M.G.); 2 School of Chemistry, Beihang University, Beijing 100191, China; lidiansen@buaa.edu.cn

**Keywords:** short carbon fiber, PLA, composite, flexural properties, failure mechanisms

## Abstract

Recently, short-fiber-reinforced thermoplastic composites (SFRTPCs) have been playing a more and more crucial role in the application of automotive interior materials due to their advantages of low density and environmental resistance properties. However, their relevant mechanical properties need to be optimized. Previous investigations revealed that the surface modification of fibers is useful to improve their mechanical properties. In this work, carbon fiber (CF)-reinforced polylactic acid (PLA) composites modified with MXene and graphene oxide (GO) were prepared by twin-screw extrusion and injection molding methods. Short CF was firstly modified with polyetherimide (PEI), then different weight ratios of MXene-GO (1:1) were subsequently modified on PEI-CF. Finally, the flexural properties and failure mechanisms were analyzed. The results showed that MXene-GO was successfully coated on CF surface, and the flexural strength and modulus of CF-PEI-MXene-GO-reinforced PLA (CF-PEI-MG/PLA) composite were improved compared to that of CF/PLA composite. In addition, the fracture sections of the composites were flat and white, and the fibers bonded well with PLA for CF-PEI-0.1MG/PLA composite compared to CF/PLA composite. The present study could provide a reference for further improving the mechanical performance of PLA-related composites.

## 1. Introduction

The energy crisis has created a strong demand for lightweight structural materials in various industries. Compared with traditional thermosetting composites, thermoplastic composites produce fewer harmful chemicals, have no curing stage and possess good recyclability, weldability and high production capacity [1,2]. Meanwhile, short-fiber-reinforced thermoplastic composites (SFRTPCs) have been used in a variety of industrial applications, such as automotive, aerospace, construction and electronic packaging, due to their advantages with low density, high strength and good environmental resistance properties [3,4].

Recently, polylactic acid (PLA), which is a new type of biodegradable thermoplastic made from starch derived from renewable plant sources, has attracted great attention [5,6]. The thermomechanical properties of PLA are excellent, with a tensile strength of about 50 MPa, melting point of 180 °C and glass transition temperature of 60 °C [7]. However, there are some disadvantages of PLA, such as poor processability, low impact strength and low heat distortion temperature [8]. It was reported that the mechanical properties of thermoplastic could be improved by adding reinforcing agents, such as fibers, glass and carbon [9,10,11], etc. Chen et al. [12] revealed that tensile strength and modulus of PLA with 30 wt% recycled carbon fiber (CF) composite increased by 73% and 438%, and flexural strength and modulus increased by 53% and 400% compared to those of neat PLA. Nadondu et al. [9] added 10 wt% CF and 20 wt% durian skin fiber into PLA (70 wt%) and found flexural strength and modulus of PLA were improved. Chen et al. [13] found that tensile strength of optimal Kenaf fibers/MWCNT/PLA composite increased by 58%, and impact strength increased by 113% compared to those of bare PLA.

In addition, it has been found that the modification of CF could also influence the mechanical properties of FRTPCs, based on the improvement of the interfacial bonding between fiber and matrix [14]. Recently, numerous studies were conducted to construct interlocking and interfacial bonding between fiber and matrix [15,16]. Xu et al. [17] found that flexural strength, flexural modulus and interlaminar shear strength of CF/poly(ether ether ketone) (CF/PEEK) composites increased when carbon fibers were modified with polyetherimide (PEI), MXene and carbon nanotube (CNT). Among various modified particles and agents, polymer coatings have attracted a great deal of attention due to their advantage of enhanced interfacial bonding on the surface of the fibers [18,19]. PEI has been used as one of the surface-modification polymers due to its excellent chemical amino groups, exceptional thermal stability and outstanding mechanical properties [20]. Thus, to improve the mechanical properties of PLA, the modification of reinforcement of fibers could be conducted to further broaden the potential application of PLA in high-performance structural areas.

By now, there are still some key technical problems to be solved in the application of SFRTPCs. Firstly, it is difficult to distribute CF uniformly in the matrix material, and interfacial bonding of fiber/matrix is poor. Secondly, the performance is not guaranteed. For example, PLA-related composites could be prepared by various technologies, such as extrusion, injection molding, film drawing, 3D printing, etc. The preparation of SFRTPCs has mainly focused on the 3D printing method, represented by fused deposition modeling, in which complex structures can be fabricated with a layer-by-layer buildup process [21,22]. Meanwhile, other preparation technologies have also been investigated by researchers. Twin-screw extrusion technology can provide uniform mixing, where the polymer material is heated to a molten state by continuous rotation of the screw, which ensures the quality and consistency of the final product [23]. Pérez-Fonseca et al. [24] used a twin-screw extrusion machine to prepare biodegradable polylactic acid/polyhydroxybutyrate (PLA/PHB) composites. Simultaneously, injection molding method can be used to manufacture structures with specially designed shapes and sizes [25]. Kumar et al. [26], using an injection molding technique, prepared a kenaf fiber and polylactic acid (KF/PLA) composite. Therefore, to broaden the development and application field of PLA-related composites, more manufacturing technologies need to be developed.

In this paper, modified short-CF-reinforced PLA (CF/PLA) composites were prepared based on twin-screw extrusion technology and an injection molding method, where CF was modified with MXene and GO. In addition, the flexural properties of their relevant composites were investigated and the failure mechanisms were analyzed. Short CF was initially grafted with PEI to introduce amino groups on the CF surface, then different weight ratios of MXene-GO (1:1) were subsequently coated on PEI-CF. The findings of this work provide a reference for further investigation of the mechanical performance of PLA-related composites.

## 2. Materials and Methods

### 2.1. Materials

CF fabrics were purchased from Easycomposites Technology Co., Ltd. (Beijing, China); PLA (6060D) was obtained from Nature Works (Minneapolis, MN, USA); HCl (36.46%) was supplied by Beijing Chemical Plant (Beijing, China); Lithium fluoride (LiF, AR) was provided by Shanghai Maclin Biochemical Technology Co., Ltd. (Shanghai, China); Ti_3_AlC_2_ (400 mesh, 98%) was bought from Jilin Yiyi Technology Co., Ltd. (Changchun, China); Nitric acid (65.0–68.0%) was purchased from Sinopharm Group Chemical Reagent Co., Ltd. (Shanghai, China); polyetherimide (600, 99%) was obtained from Shanghai Aladdin Biochemical Technology Co., Ltd. (Shanghai, China); GO (2 mg/mL) was obtained from Nanjing Xianfeng Nanomaterial Technology (Nanjing, China). All the chemicals were used as received.

### 2.2. Preparation of MXene Nanosheets

MXene nanosheets were prepared based on the method of [27,28]. Specifically, 2.0 g LiF was added into 40 mL 9 M HCI solution in a plastic beaker. Then, 2.0 g Ti_3_AlC_2_ powder was slowly added into the mixed solution and kept at 35 °C for 24 h under continuous magnetic stirring. After the reaction, the precipitate was repeatedly washed with deionized water (DI) and centrifuged at 3500 rpm for 5 min until the pH of the supernatant reached 6. The collected precipitate was then re-dispersed into DI and ultrasonically treated under Ar gas for 1 h. Finally, the above mixture was centrifuged at 3500 rpm for 1 h and the dark green supernatant with MXene nanosheet was obtained.

### 2.3. Preparation of Modified CF

**Figure 1:**
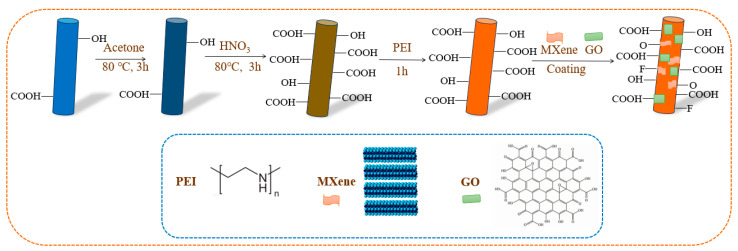
The diagram of surface modification process of CF.

Figure 1 shows the modification process of CF. In order to remove the impurities on the fiber surface, CF was initially placed in acetone at 80 °C for 3 h, then was washed with deionized water (DI) for several times. After drying at 60 °C in the oven, the desized CF was obtained. Secondly, the desized CF was placed in concentrated nitric acid solution at 80 °C for 3 h. After reaction, the CF was repeatedly washed with DI. Then, it was dried at 60 °C to obtain carboxylated CF. Thirdly, the carboxylated CF was immersed in PEI solution (1 mg/mL) for 1 h, and the dried CF, called PEI-CF, was obtained. Finally, PEI-CF was modified with different mass concentrations of MXene-GO (mass ratio of 1:1) solution for 2 h at room temperature, and the concentrations of which were 0.05 wt%, 0.1 wt% and 0.2 wt%, respectively. The dried modified CFs, named CF-PEI-0.05MG, CF-PEI-0.1MG and CF-PEI-0.2MG, were obtained, respectively.

### 2.4. Preparation of Modified CF/PLA Composite

**Figure 2:**
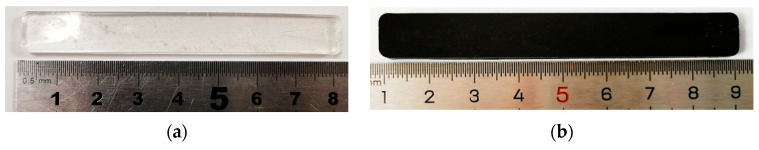
The flexural specimen of (**a**) pure PLA and (**b**) CF/PLA composite.

The dried CFs was cut into short CFs, and the length of which was about 5 mm. Subsequently, the mixture of short CFs and PLA with weight ratio of 1:30 was put into the twin-screw extruder to obtain evenly mixed CF and PLA, and extruded to a uniform long strip at 185 °C. The same procedure was performed for the CF-PEI-0.05MG, CF-PEI-0.1MG and CF-PEI-0.2MG with short CF. Then, the strips of CF/PLA, CF-PEI/PLA, CF-0.05wt%MG/PLA, CF-0.1wt%MG/PLA and CF-0.2wt%MG/PLA were cut into uniform size masterbatches, respectively. Finally, the masterbatches were dried in a vacuum-drying oven for 4 h at 80 °C and then injected into the injection molding machine to obtain flexural specimens to further investigate their flexural properties. During the injection process, the temperature of the pressing zone was set to 55 °C, the injection molding zone was 175 °C and the barrel was 185 °C. In addition, the rotating speed was set as 36–40 rpm/min and the injection pressure was set to 0.6 MPa. Meanwhile, the mold temperature was at room temperature, the pressure holding time was 10 s and the circulation time of the masterbatches was 3 min. After injection molding, the flexural samples could be obtained immediately after demolding. Samples (80 mm × 10 mm × 4 mm) were prepared for each kind of composite, and the specimen morphologies are shown in Figure 2. It can be seen that after modification with CF, the color of PLA was changed to black.

### 2.5. Characterizations

The surface morphologies of modified CF and their fracture specimens were characterized by scanning electron microscopy (SEM, Hitachi S-4700, Tokyo, Japan). The flexural specimens were tested according to ASTM D790 standard [29] on the computerized electronic universal testing machine (Jinan Tianchen Testing Machine Manufacturing Co. Ltd., Jinan, China, with the maximum load of 20 KN) at the speed of 0.5 mm/min, and the flexural span was set as 64 mm. In addition, three samples were tested at the same experimental condition.

The flexural strength of the specimen was calculated as follows [30]:

(1) $$\sigma_{f}=\frac{3pL}{2bh^{2}}$$

where $\sigma_{f}$ is bending strength (MPa), *p* is failure load (N), *L* is span (mm), *b* is width of specimen (mm) and *h* is the thickness of specimen (mm);

The bending modulus of the specimen was calculated according to the following formula [30]:

(2) $$E_{f}=\frac{∆PL^{3}}{4∆Sbh^{3}}$$

where $E_{f}$ is the flexural elastic modulus (GPa), ∆*P* is the load increment (N) of the initial straight segment on the load-deflection curve and ∆*S* is the deflection increment (mm) corresponding to the load increment ∆*P*.

The flexural strain of the specimen was calculated as follows [31]:

(3) $$\varepsilon =\frac{6Sh}{L^{2}}$$

## 3. Results and Discussion

### 3.1. Surface Morphology of CFs

**Figure 3:**
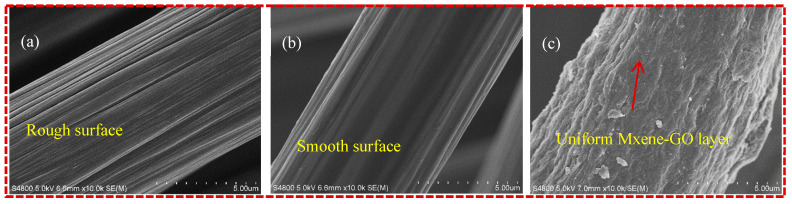
Surface morphologies of (**a**) CF, (**b**) CF-PEI and (**c**) CF-PEI-0.1MG.

Figure 3 provides surface morphologies of CF, CF-PEI and CF-PEI-0.1MG. It can be observed that grooves were uniformly distributed along the fiber axis on the surface of CF in Figure 3a. After grafting with the PEI layer, the grooves were partially filled, the surface of fiber became smooth and the grooves were not obvious compared to original CF (Figure 3b). For CF-PEI-0.1MG in Figure 3c, the convex grooves were thoroughly covered with MXene-GO on the surface of the fiber, indicating the effective introduction of MXene and GO nanoparticles.

### 3.2. Flexural Strain–Stress Curves

**Figure 4:**
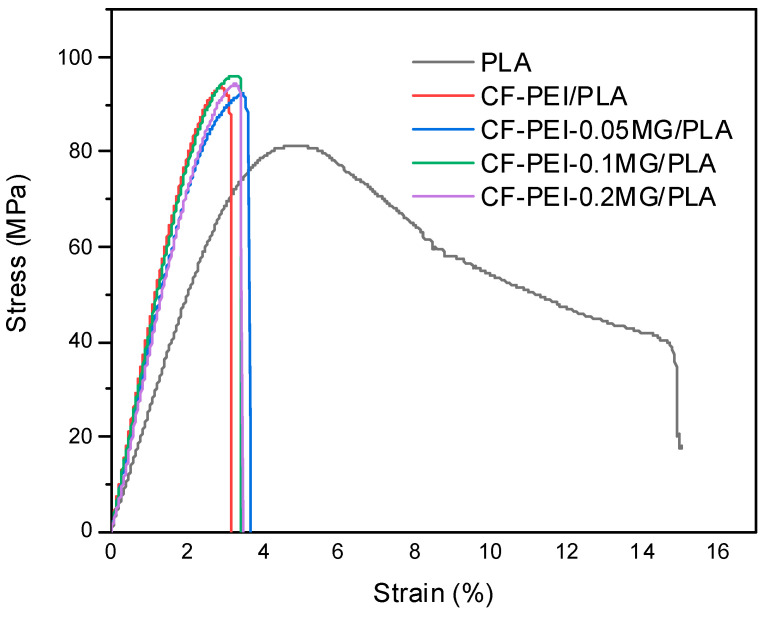
Flexural stress–strain curve of PLA, CF-PEI/PLA, CF-PEI-0.05MG/PLA, CF-PEI-0.1MG/PLA and CF-PEI-0.2MG/PLA composites.

Figure 4 shows the flexural stress–strain curves of PLA, CF-PEI/PLA, CF-PEI-0.05MG/PLA, CF-PEI-0.1MG/PLA and CF-PEI-0.2MG/PLA composites. It can be seen that the curves grew linearly at the initial stage. Then, they grew steadily up to the maximum strength. Finally, they dropped sharply and directly for CF-PEI/PLA, CF-PEI-0.05MG/PLA, CF-PEI-0.1MG/PLA and CF-PEI-0.2MG/PLA composites, with brittle failure characteristics. The changes were different from that of pure PLA, which dropped gradually, showing plastic fracture feature. In addition, they were also different from those of fiber-reinforced PLA in [32,33]. The reason might be that the length of fibers was too short to effectively achieve the purpose of reinforcement of PLA. Therefore, they descended directly and quickly, rather than with the feature of a stepped drop.

### 3.3. Flexural Mechanical Properties

**Figure 5:**
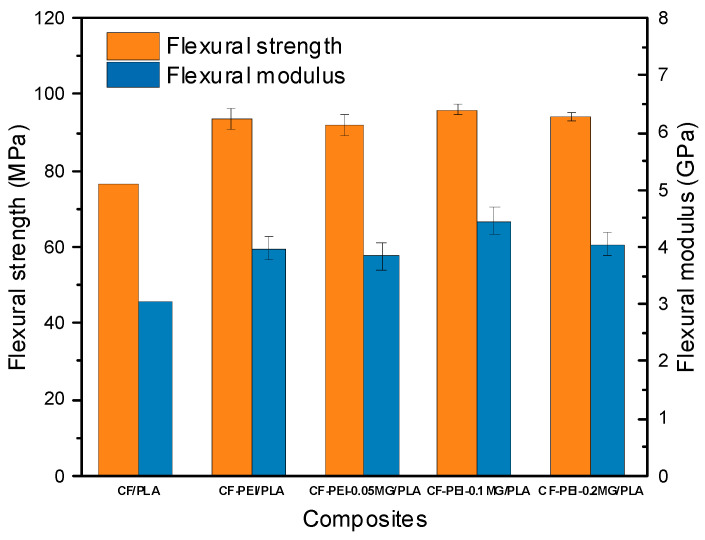
Flexural strength and modulus of CF/PLA, CF-PEI-0.05MG/PLA, CF-PEI-0.1MG/PLA and CF-PEI-0.2MG/PLA composites.

Figure 5 and Table 1 give the flexural strength and modulus of CF/PLA, CF-PEI-0.05MG/PLA, CF-PEI-0.1MG/PLA and CF-PEI-0.2MG/PLA composites. Comparing to the flexural strength and modulus of CF/PLA composite [33], the strength of CF-PEI-0.05MG/PLA, CF-PEI-0.1MG/PLA and CF-PEI-0.2MG/PLA composites were, respectively, improved by 22.72%, 20.69%, 25.93% and 23.52%, and the modulus were separately improved by 30.07%, 25.82%, 45.42% and 32.35%. The mechanical properties of CF-PEI-0.1MG/PLA were the best, and the maximum flexural strength was 96.12 MPa and flexural modulus was 4.45 GPa. The reason might be that active amine groups were effectively introduced on the surface of CF by PEI, which could take part in the reaction with PLA and improved the interfacial bonding of fiber/PLA [34,35]. For PEI-MG-modified CF/PLA composites, MXene and GO may have served as an anchor between fiber and PLA, further increasing the mechanical interlocking of CF/PLA. As a result, the flexible PEI and rigid MG constructed a gradient interface layer, effectively improving the flexural properties of modified CF/PLA [36,37]. However, after modification with a higher content of MG, the flexural properties were slightly decreased due to decreased interfacial bonding between fiber and PLA. On the other hand, the improvement of flexural properties of CF/PLA composite was limited due to multiple cuts of CF to prepare flexural specimens; consequently, the length of CF was too short to effectively transfer the load from fibers to matrix, which could not highly improve mechanical properties of CF/PLA composites. In addition, it might have been due to the fact that the CF content was low. Therefore, PEI and MG could effectively improve the properties of CF/PLA composites reinforced with short carbon fibers to a certain extent.

### 3.4. Flexural Failure Analysis

**Figure 6:**
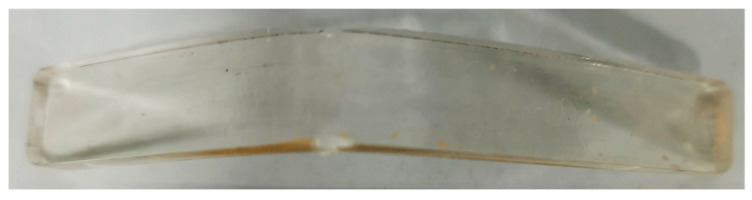
Macroscopic flexural fracture failure of PLA.

Figure 6 gives macroscopic flexural fracture failure of PLA. It can be seen that the PLA showed a plastic fracture feature after flexural load, which was in accordance with the PLA stress–strain curve in Figure 4.

**Figure 7:**
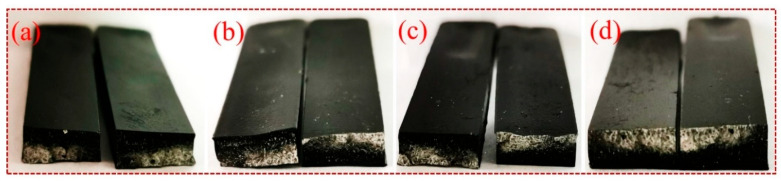
Macroscopic flexural fracture failure of composites (**a**) CF-PEI/PLA, (**b**) CF-PEI-0.05MG/PLA, (**c**) CF-PEI-0.1MG/PLA and (**d**) CF-PEI-0.2MG/PLA.

**Figure 8:**
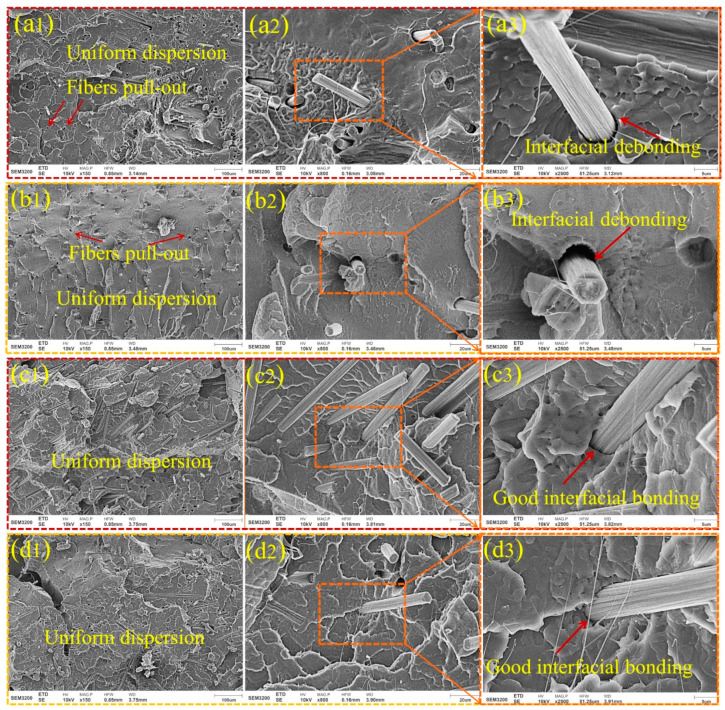
Macroscopic flexural fracture failure modes of composites with magnification of ×150, ×800 and ×2500, respectively. (**a1**–**a3**) CF-PEI/PLA, (**b1**–**b3**) CF-PEI-0.05MG/PLA, (**c1**–**c3**) CF-PEI-0.1MG/PLA and (**d1**–**d3**) CF-PEI-0.2MG/PLA.

Figure 7 and Figure 8 show the macro and micro flexural fracture failure modes of CF-PEI/PLA, CF-PEI-0.05MG/PLA, CF-PEI-0.1MG/PLA and CF-PEI-0.2MG/PLA composites. As can be seen from the fracture section diagram in Figure 7, the fracture sections of the composites were flat and white, showing a typical stress whitening phenomenon. In addition, the composites became black with the introduction of CF. Meanwhile, the bended specimens were thoroughly broken compared to those in [33], which was due to the fibers that were cut too short and could not effectively reinforce the PLA as continuous fibers.

Figure 8 exhibits SEM photographs of flexural fracture surfaces of different kinds of CF/PLA composites. Matrix cracking and fiber pull-out features resulted in the composite’s failure. It could be seen from Figure 8(a1,b1) that under flexural load, a large number of small pores were left because the short fibers were pulled out, indicating that interfacial bonding of CF-PEI/PLA and CF-PEI-0.05MG/PLA was poor. At the same time, the pore distribution was uniform, indicating that the fibers were evenly distrubted in PLA after multiple cuttings and mixings of CF and PLA. In Figure 8(a2,a3,b2,b3), the interfacial debonding features of fibers can be distinctively seen, which denotes that PEI and little content of MXene-GO could not effectively improve the interfacial bonding between fiber and PLA. In contrast, in Figure 8c,d, the amount of pull-out features of fibers decreased remarkably. In particular, in Figure 8c1, the fibers were still bound well to PLA even after flexural load, where fibers were still evenly imbeded in PLA, which verified good infterfcial bonding of CF-PEI-0.1MG/PLA (Figure 8(c2,c3)), resulting in higher mechanical properties. In Figure 8d, it can be observed that there was still a certain amount of fibers imbeded in PLA after fracture, showing that the interfical bonding of CF-PEI-0.2MG/PLA was relatively better compared to those of CF/PLA and CF-PEI-0.05MG/PLA composites. Therefore, proper content of MXene-GO modification of CF could improve the mechanical properties of PLA.

**Figure 9:**
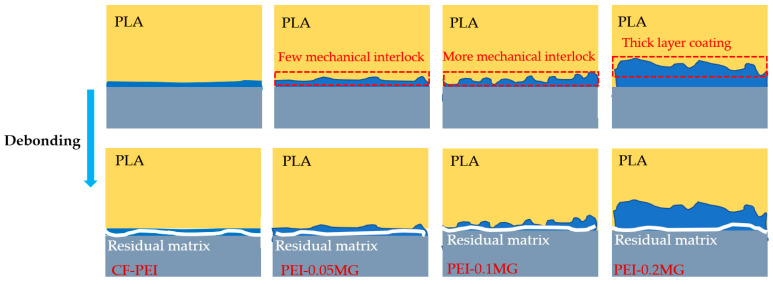
Schematic diagram of the fracture mechanisms of PLA debonding from modified CFs.

The schematic diagram of interfacial fracture mechanisms of modified CF-reinforced PLA composites is given in Figure 9. A relatively poor interfacial bonding feature between CF-PEI and PLA was observed due to the smooth surface of CF. In this case, the poor interfacial bonding of fiber/matrix could not effectively and quickly transfer the stress, which subsequently caused the CF-PEI/PLA composite to fail, with a large crack between fiber and matrix. After CF-PEI grafted with 0.05MG, the amino groups on the surface of CF-PEI reacted with the functional groups of MG, which improved the chemical bonding of the interphase. In addition, few mechanical interlock phenomena occurred, which further enhanced the interfacial bonding of CF/PLA composite. The enhanced interfacial bonding slightly enabled the relatively effective transfer of stress among the interphase, which slightly improved the mechanical properties of the CF/PLA composite. Moreover, after further constructing the PEI-0.1MG microstructures on the CF surface, more mechanical interlocking on the interphase was greatly enhanced, which finally resulted in great improvement of mechanical properties. However, when grafted with MG of 0.2 wt%, the thick layer of interphase was constructed and the rigid interfacial layer led to the mechanical properties of CF/PLA composite being unable to be more highly improved. In a word, the constructed PEI-MG microstructures with 0.1 wt% MG could form a strong mechanical interlocking between fibers and the PLA matrix, resulting in an enhanced interfacial bonding.

## 4. Conclusions

In this work, CF-PEI-MG/PLA composites were successfully prepared by twin-screw extrusion and injection molding methods, and CF was uniformly distributed in PLA. PEI and MXene-GO were effectively coated on the surface of CF, indicating the successful modification of CF. After modification, the flexural strength of the CF-PEI-0.1MG/PLA composite was 96.12 MPa, which was improved by 25.93% compared to that of the CF/PLA composite, and the modulus reached 4.45 GPa, which was enhanced by 45.42%. In addition, for the failure mechanism of the composites, it was shown that the fracture sections were flat and white, showing a typical stress whitening phenomenon. Matrix cracking and fiber pull-out features finally resulted in the failure of the composite since the fibers were too short. Meanwhile, fibers were still uniformly distributed well in PLA for CF-PEI-0.1MG/PLA composite after failure, and the interfacial bonding of fiber and PLA was tightly bonded since more mechanical interlock was constructed. However, the multiple cuts of CFs could only enhance the mechanical properties of CF/PLA composite to a limited extent, as expected. In future investigations, the length of CFs will be designed to the maximum to further improve the mechanical properties of PLA. It is expected that the present study provides a useful reference for preparing high mechanical performance of SFRTPCs.

## Figures and Tables

**Table 1:** Flexural strength and modulus of different CF/PLA composites.

Composites	Flexural Strength (MPa)	Flexural Modulus (GPa)	Fracture Strain (%)	References
CF/PLA	76.33	3.06	-	[33]
CF-PEI/PLA	93.67	3.98	3.01	In this work
CF-PEI-0.05MG/PLA	92.12	3.85	3.48	In this work
CF-PEI-0.1MG/PLA	96.12	4.45	3.41	In this work
CF-PEI-0.2MG/PLA	94.28	4.05	3.31	In this work

## Data Availability

All data included in this study are available upon request by contact with the corresponding author due to privacy.
